# Overweight predisposes to microvascular obstruction: insights from the TATORT-NSTEMI trial

**DOI:** 10.1007/s10554-026-03606-y

**Published:** 2026-01-19

**Authors:** Felix Troger, Ingo Eitel, Roza Saraei, Thomas Stiermaier, Michael Böhm, Bernward Lauer, P. Christian Schulze, Tobias Geisler, Leonhard Bruch, Norbert Klein, Uwe Zeymer, Agnes Mayr, Maria Buske, Steffen Desch, Holger Thiele, Hans-Josef Feistritzer

**Affiliations:** 1 University Clinic of Radiology, Medical University of Innsbruck, Innsbruck, Austria; 2 Department of Cardiology, Angiology and Intensive Care Medicine, Heart Center Lübeck, University, University Hospital Schleswig-Holstein, Lübeck, Germany; 3 German Center for Cardiovascular Research (DZHK), partner site Hamburg/Kiel/Lübeck/Greifswald, Lübeck, Germany; 4 Department of Internal Medicine III, University of Saarland, Homburg, Germany; 5 Department of Cardiology, University Hospital Jena, Jena, Germany; 6 Department of Cardiology/Cardiovascular Medicine, University of Tübingen, Tübingen, Germany; 7 Department of Internal Medicine, Unfallkrankenhaus Berlin, Berlin, Germany; 8 Klinik für Kardiologie und Internistische Intensivmedizin, Klinikum St. Georg, Leipzig, Germany; 9 Stiftung Institut für Herzinfarktforschung, Ludwigshafen, Germany; 10 Cardiology, Hemostaseology, and Medical Intensive Care, Medical Centre Mannheim, Medical Faculty Mannheim, Heidelberg University, German Center for Cardiovascular Research (DZHK), Partner Site Heidelberg/Mannheim, Mannheim, Germany; 11 Department of Cardiology and Angiology, Faculty of Medicine, University Heart Center Freiburg–Bad Krozingen, University of Freiburg, Freiburg, Germany; 12 Department of Internal Medicine/Cardiology, Heart Center Leipzig at Leipzig University, Strümpellstr. 39, 04289 Leipzig, Germany

**Keywords:** Body mass index, Microvascular obstruction, Myocardial infarction, Cardiac magnetic resonance imaging

## Abstract

Microvascular obstruction (MVO) is a phenomenon associated with worse outcome after acute myocardial infarction. While some studies suggested a benefit of an increased body mass index (BMI) in terms of MVO occurrence in ST-elevation myocardial infarction (STEMI), there are currently no data available on the influence of overweight on the development of MVO in Non-STEMI (NSTEMI). Thus, the aim of this study was to assess the association between MVO and BMI in NSTEMI patients. This study investigated a sub-cohort of the TATORT-NSTEMI trial. Overall, 354 patients were included with a median age of 68 years (25% women). Cardiac magnetic resonance imaging (CMR) was performed within four days after the index event. MVO was defined as hypointense core within the infarcted area in late-enhancement sequences. MVO occurred in 97 patients (27%) and patients with MVO had a significantly higher BMI (28.4 kg/m², interquartile range (IQR) 26.1–31.1 vs. 27.3 kg/m², IQR 24.8–30.3, *p* = 0.024). Dichotomized at 25.6 kg/m² (Youden-index), patients with a BMI above that threshold showed significantly more often MVO (33% vs. 15%, *p* < 0.001). In logistic regression analysis, BMI > 25.6 kg/m² was a significant predictor of MVO, independent of traditional cardiovascular risk factors. In the current study, an increased BMI has been associated with MVO after NSTEMI. Further, overweight and especially a BMI above 25.6 kg/m² were independent predictors of MVO in these patients, challenging the so-called “obesity paradox”. Lastly, further research on the connection between body weight and microvascular damage in myocardial infarction is needed.

## Introduction

Microvascular obstruction (MVO) following myocardial infarction is regarded a decisive predictor of adverse remodeling [1], heart failure [2], and mortality [3]. The pathogenesis of MVO is a compound of several factors, primarily consisting of reperfusion injury and distal embolization, and secondarily of microvascular compression due to edema [4]. Furthermore, diabetes mellitus is assumed to play a crucial role in terms of comorbidities [5]. The role of overweight, e.g., defined by the body mass index (BMI), in the development of MVO is currently not fully understood. Although overweight is regarded a major clinical risk factor for adverse outcomes after myocardial infarction [6], there are studies suggesting that it could be a protective factor in terms of MVO [7]. Contrary, another study showed that overweight might enhance the negative effects of diabetes regarding microvascular injury after acute myocardial infarction [8]. While most of these studies concentrated on ST-elevation myocardial infarction (STEMI), there are limited data available concerning the effects of overweight on the development of MVO in patients suffering myocardial infarction without ST-elevation (NSTEMI). Obesity is associated with an increased risk of NSTEMI and an earlier onset compared to normal-weight patients [9]. Still, also in NSTEMI patients an increased weight might have potential protective benefits, such as lower mortality and fewer complications [9, 10]. This highlights a complex relation of body weight and outcome in NSTEMI. Thus, this current study aimed to investigate the association of MVO assessed by cardiovascular magnetic resonance imaging (CMR) with overweight in NSTEMI patients.

## Methods

### Study population

The currently investigated population represented a sub-cohort from the TATORT-NSTEMI trial (Thrombus Aspiration in Thrombus Containing Culprit Lesions in NSTEMI), a multicenter, randomized, open-label trial comparing percutaneous coronary intervention (PCI) with aspiration thrombectomy versus conventional PCI in NSTEMI patients with thrombus-containing culprit lesions. Design, objectives and main results have been published previously [11, 12]. Prior to study inclusion, written informed consent was given by all participants. The study received approval by the local research ethics committee.In brief, inclusion criteria were defined as: (a) ischemic symptoms lasting > 20 min; (b) last symptoms occurring < 72 h before randomization; (c) cardiac troponin T levels above the 99th percentile; (d) thrombus containing culprit lesion according to Thrombolysis in Myocardial Infarction (TIMI) thrombus grades 2–5; and (e) intended early PCI. Posterior ECG leads were routinely performed to exclude posterior STEMI.

Exclusion criteria were diagnosis of STEMI, cardiogenic shock, no identifiable culprit lesion or a coronary morphology ineligible for thrombectomy, indication for acute bypass surgery, age < 18 and > 90 years, contraindications to treatment with heparin, aspirin or thienopyridines, pregnancy, current participation in another trial, severe comorbidity with life expectancy < 6 months, and contraindications to CMR.

Per study protocol, eligible patients were randomized to either aspiration thrombectomy prior to PCI or conventional PCI in a 1:1 ratio. Thrombectomy was performed using a commercially available aspiration catheter (Eliminate^®^, Terumo Europe, Leuven, Belgium). Except for thrombectomy, all other therapeutic procedures were guideline-directed and identical in both treatment arms.

### Cardiovascular magnetic resonance imaging

**Fig. 1:**
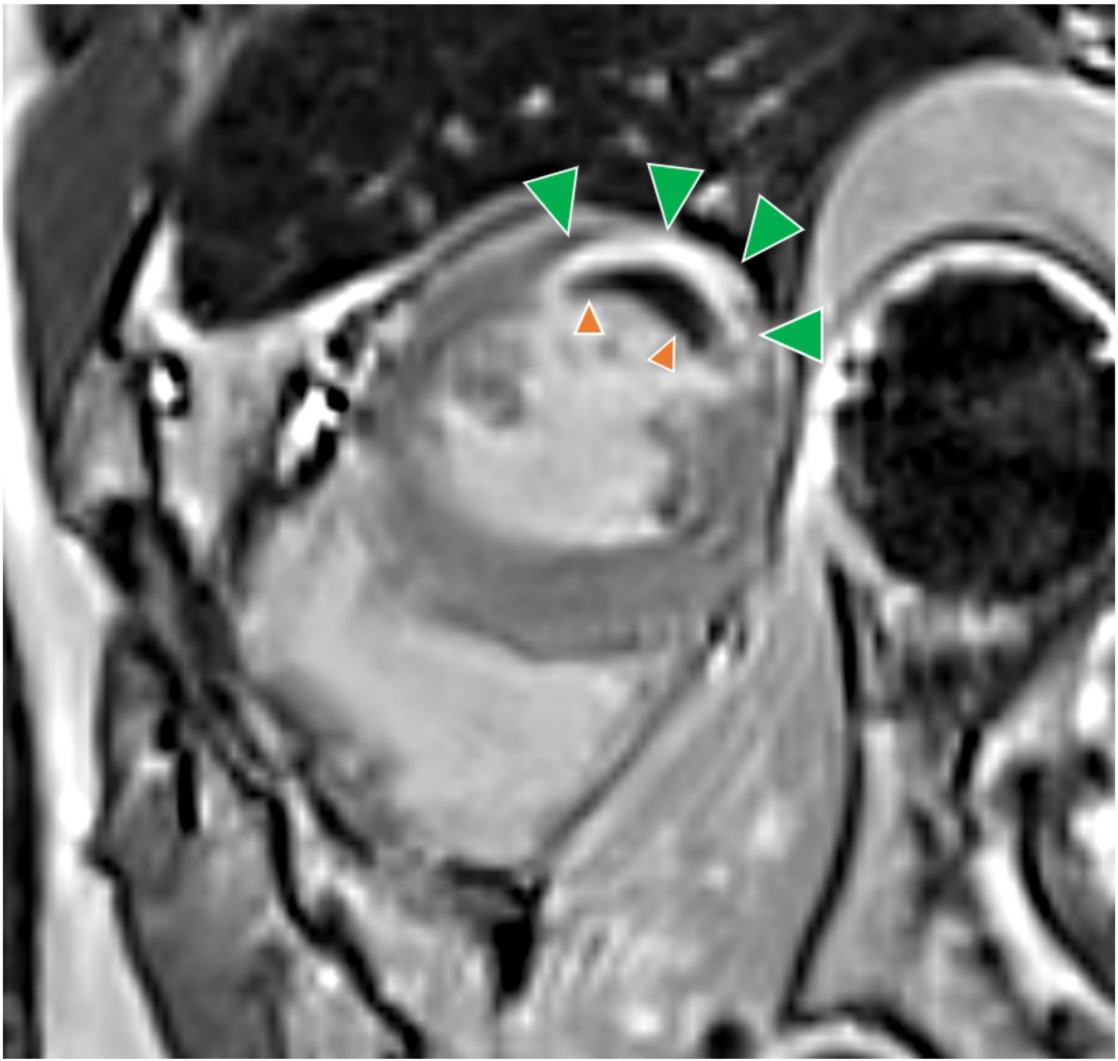
Short-axis late-enhancement image of a 55-year old NSTEMI patient with a body mass index of 31 kg/m², with transmural infarction of the anterolateral wall (green arrowheads) and a large subendocardial MVO component (orange arrowheads). *MVO: microvascular obstruction; NSTEMI: non-ST-elevation myocardial infarction*

CMR was performed on a 1.5- or 3.0-T scanner on days 1 to 4 after randomization using a standard scan protocol, as described previously [11]. In brief, late MVO and infarct size were assessed using late-enhancement short-axis images covering the whole left ventricle (LV) 10 to 20 min after injection of gadolinium-containing contrast agent. An inversion recovery turbo gradient echo sequence was used for image acquisition. Assessment of LV function and volumes was performed using a standard steady-state free precession technique acquiring short-axis slices from base to apex. LV ejection fraction (EF) was calculated from the short-axis functional views. The blinded observers used certified CMR evaluation software (cvi42, Circle Cardiovascular Imaging Inc., Calgary, Canada). Semiautomated computer-aided threshold detection was applied to detect MVO and infarcted myocardium, as previously described [12–15], whereby MVO was defined as hypointense core within the hyperintense infarcted area on late-enhancement sequences, as depicted in Fig. 1.

### Statistical analysis

SPSS Statistics 26.0 (IBM, Armonk, NY, USA) was used for statistical analyses. All results for continuous variables are expressed as medians with corresponding interquartile range (IQR), categorical variables as absolute numbers and percentages. Differences in continuous variables were tested using Mann-Whitney-Test. Differences in categorical variables between groups were tested by Chi-square test.

A post-hoc receiver operating characteristic (ROC) analysis was performed to determine an optimized BMI threshold for predicting MVO, applying the Youden-index. Binary logistic regression was performed to evaluate independent markers for MVO. Thereby, univariable analysis was performed on all parameters showing significant differences between patients with and without MVO; then, in addition to dichotomized BMI, variables with a p-value < 0.1 in univariable analysis were included in the multivariable models. To increase interpretability, metric variables were stratified into quartiles for logistic regression analyses. A p-value < 0.05 was considered statistically significant.

## Results

### Baseline patient characteristics

Overall, 354 patients with revascularized NSTEMI were included in this current study, comprising all patients of the TATORT-NSTEMI trial that were enrolled at the primary investigator site (Heart Center Leipzig at Leipzig University). The median age was 68 years (IQR: 57–74) and 88 patients were women (25%). MVO was detected in 97 patients (27%). A detailed list of baseline characteristics is shown in Table 1.

**Table 1:** Baseline patient characteristics. BMI: body mass index; EDV: end-diastolic-volume, EF: ejection fraction; ESV: end-systolic volume; hs: high-sensitive; LV: left ventricular; MVO: microvascular obstruction, PCI: percutaneous coronary intervention, TIMI: thrombolysis in myocardial infarction

	Overall (*n* = 354)	No MVO (*n* = 257)	MVO (*n* = 97)	*p*-value
Age, years	68 (57–74)	70 (58–77)	61 (54–71)	**< 0.001**
Female, n (%)	88 (25)	72 (28)	16 (16)	**0.025**
Body weight, kg	82 (74–93)	81 (71–92)	86 (79–96)	**< 0.001**
BMI, kg/m²	27.7 (25.1–30.4)	27.3 (24.8–30.3)	28.4 (26.1–31.1)	**0.024**
<18.5	2 (0.5)	2 (0.8)	0 (0)	0.059
18.5–24.9	81 (23)	68 (26)	13 (13)	
25-29.9	172 (49)	115 (45)	57 (59)	
30-34.9	71 (20)	49 (19)	22 (23)	
35-39.9	21 (6)	17 (7)	4 (4)	
≥40	7 (2)	6 (2)	1 (1)	
Smokers, n (%)	138 (39)	96 (37)	42 (43)	0.306
Hypertension, n (%)	274 (77)	203 (79)	71 (73)	0.245
Diabetes, n (%)	105 (30)	85 (33)	20 (21)	**0.022**
TIMI flow pre-PCI, n(%)				
− 0	137 (39)	75 (29)	62 (64)	**< 0.001**
− 1	28 (8)	21 (8)	7 (7)	0.764
− 2	103 (29)	84 (33)	19 (20)	**0.016**
− 3	86 (24)	77 (30)	9 (9)	**< 0.001**
TIMI 3 post-PCI, n(%)	316 (89)	237 (92)	79 (81)	**0.003**
Peak hs-Troponin T, ng/ml	789 (284–2074)	599 (241–1303)	2231 (896–3708)	**< 0.001**
LV-EF, %	51 (44–57)	52 (47–59)	46 (39–55)	**< 0.001**
LV-EDV, ml	138 (114–166)	132 (110–161)	154(125–182)	**< 0.001**
LV-ESV, ml	65 (52–89)	61 (48–83)	77 (61–107)	**< 0.001**
LV myocardial mass, g	127 (110–152)	123 (106–145)	142 (123–160)	**< 0.001**
Infarct volume, ml	9 (3–21)	6 (2–15)	20 (14–30)	**< 0.001**
MVO-volume, ml	-	-	2 (1–4)	**-**

### Microvascular obstruction

Patients presenting with MVO were significantly younger (61 years, IQR: 54–71 vs. 70 years, IQR: 58–77, *p* < 0.001), more often male (84% vs. 72%, *p* = 0.025) and had a higher BMI (28.4 kg/m², IQR: 26.1–31.1 vs. 27.3 kg/m², IQR: 24.8–30.3, *p* = 0.024). Further, they suffered less often from diabetes (21% vs. 33%, *p* = 0.022). Patients with MVO had higher peak values of troponin T, lower EF and larger infarct volumes (all *p* < 0.001) as well as a significantly lower TIMI flow pre- and post-vascularizaion (*p* < 0.005 for both). There was no significant difference concerning culprit vessels (*p* = 0.683). A detailed comparison of patients with and without MVO is given in Table 1. C-reactive protein was analyzed in 105 patients and did not show a significant difference between MVO and no MVO or between patients with a BMI above and below Youden-indexed threshold (both *p* > 0.8).

**Fig. 2:**
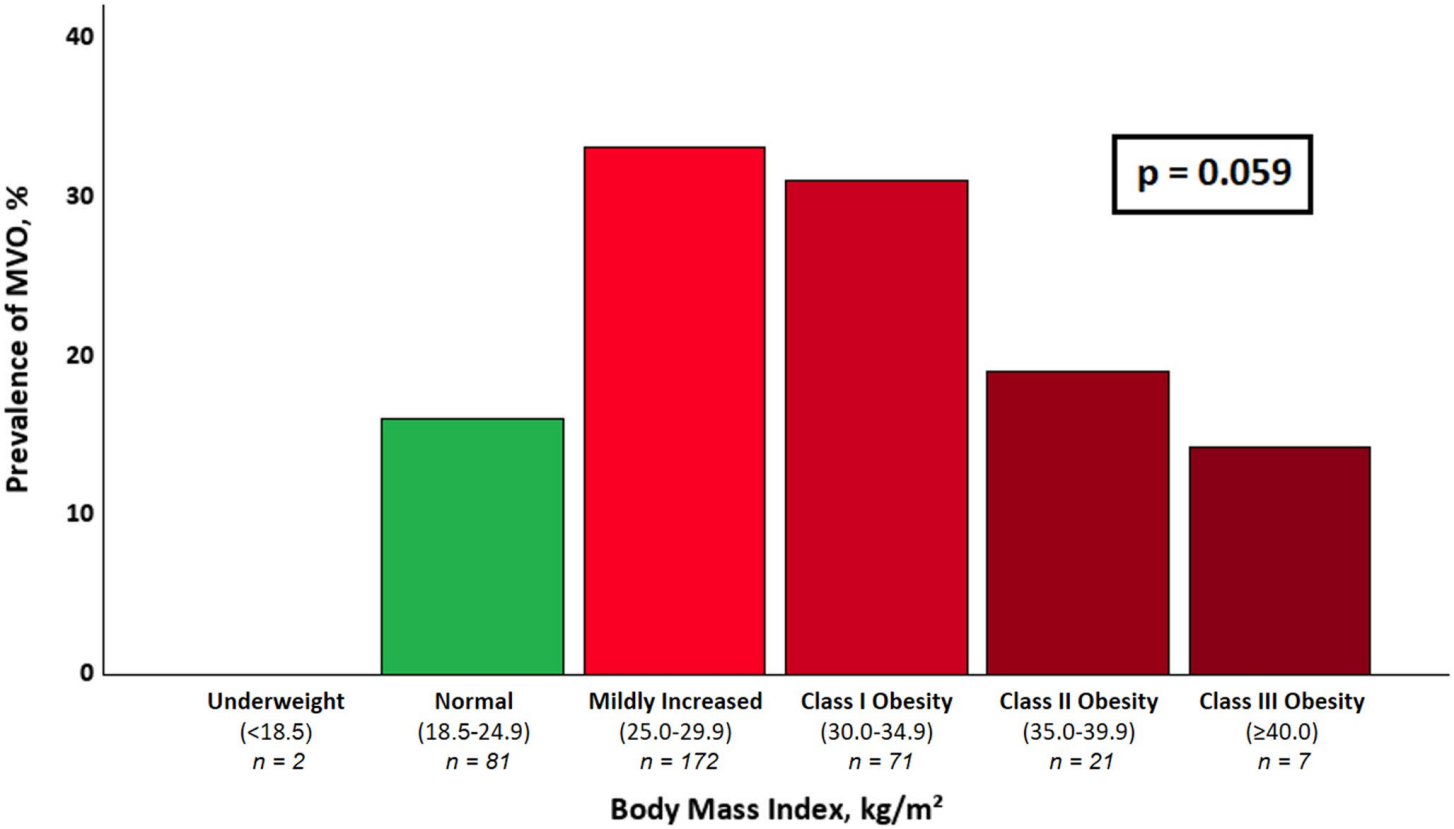
Bar chart showing the prevalence of MVO according to weight classes as stratified by the BMI. *BMI: body mass index*,* MVO: microvascular obstruction*

**Fig. 3:**
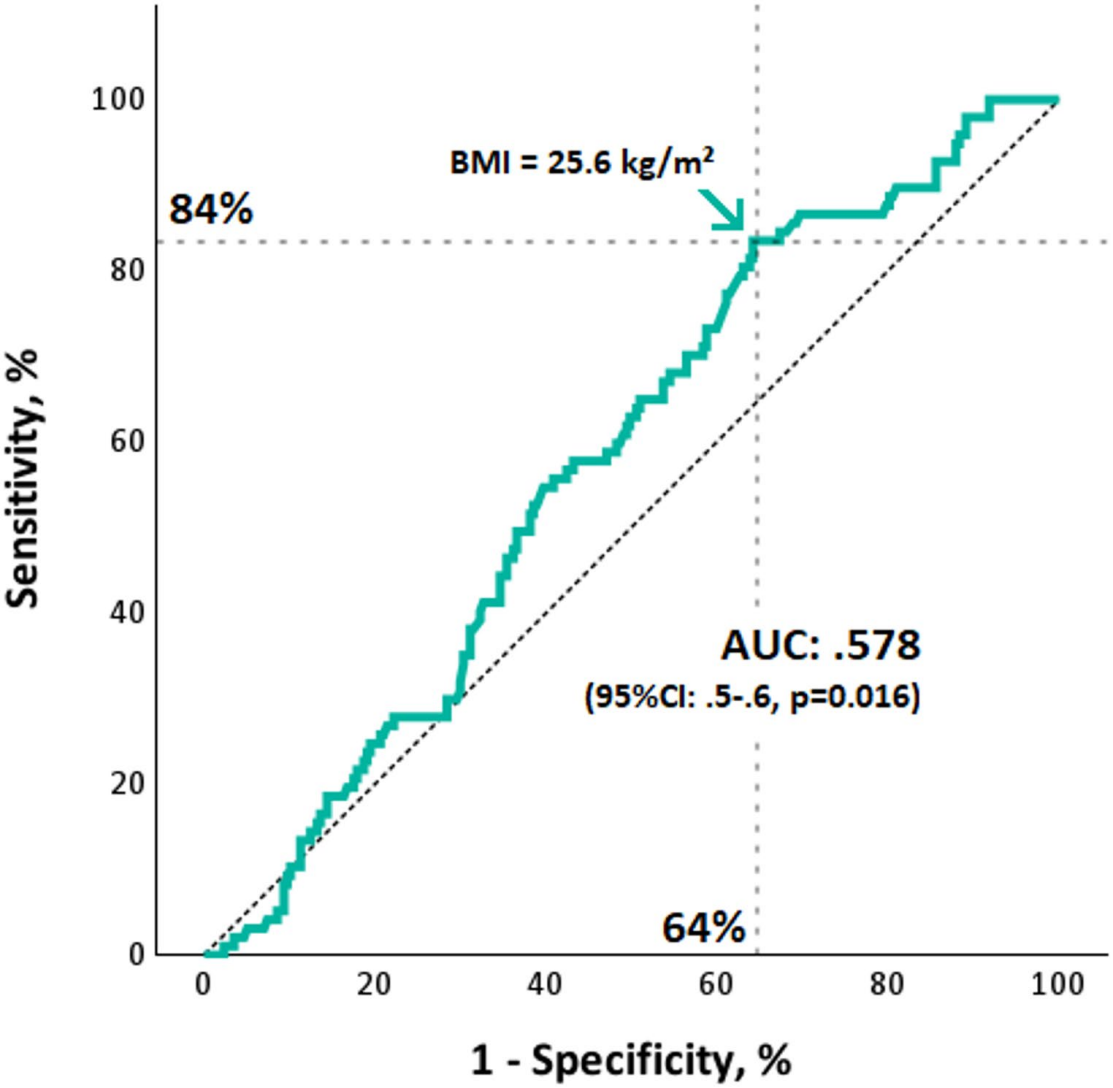
Receiver operating characteristics curve displaying sensitivity and specificity of BMI in terms of MVO presence. For this study, a threshold of 25.6 kg/m² (Youden-index) was used to dichotomize BMI. *AUC: area under the curve; BMI: body mass index; MVO: Microvascular obstruction*

BMI was analyzed as a metric variable as well as stratified into BMI classes [16] (Table 1). After dichotomizing BMI at 25.6 kg/m² (Youden-index), patients above that threshold had significantly more often MVO (33% vs. 15%, *p* < 0.001). Moreover, patients with a BMI above 25.6 kg/m² were significantly younger and had a higher end-diastolic volume and myocardial mass (all *p* < 0.05). TIMI flow pre-PCI was not significantly different between patients above and below that threshold (*p* = 0.663); on the other hand, after PCI patients with a BMI > 25.6 kg/m² had significantly more often TIMI grade 2 (10% vs. 1%, *p* = 0.003) and less often grade 3 (86% vs. 96%, *p* = 0.005), with no difference concerning grade 0 and 1. A detailed comparison of patients with a BMI above and below that threshold is shown in Table 2. There were no significant differences concerning performed thrombus aspiration between patients with and without MVO (*p* > 0.6). A bar chart displaying MVO prevalence according to different BMI classes is shown in Fig. 2. The prevalence of MVO increased from normal-weight to overweight categories, with the highest proportion observed in patients with mildly increased BMI (25–29.9 kg/m²). By tendency, a subsequent decline in the more obese range was noted (*p* = 0.059). A ROC-curve showing sensibility and specificity of BMI in terms of MVO is shown in Fig. 3.

**Table 2:** Comparison between patients with a BMI above and below the optimized threshold of 25.6 (Youden-index). BMI: body mass index; EDV: end-diastolic-volume, EF: ejection fraction; ESV: end-systolic volume; hs: high-sensitive; LV: left ventricular; MVO: microvascular obstruction, PCI: percutaneous coronary intervention, TIMI: thrombolysis in myocardial infarction

	BMI ≤ 25.6 kg/m² (*n* = 108)	BMI > 25.6 kg/m² (*n* = 246)	*p*-value
Age, years	70 (60–78)	66 (55–73)	**0.002**
Female, n (%)	34 (31)	54 (22)	0.056
Smokers, n (%)	39 (36)	99 (40)	0.463
Hypertension, n (%)	80 (74)	194 (79)	0.321
Diabetes, n (%)	27 (25)	78 (32)	0.203
TIMI 0 pre-PCI	37 (34)	100 (41)	0.256
TIMI 3 post-PCI	104 (96)	212 (86)	**0.005**
Peak hs-Troponin T, ng/ml	710 (268–1887)	892 (310–2149)	0.393
LV-EF, %	51 (44–57)	51(44–58)	0.696
LV-EDV, ml	130 (110–158)	140 (116–172)	**0.043**
LV-ESV, ml	65 (51–83)	66 (52–93)	0.298
LV myocardial mass, g	116 (100–142)	134 (113–156)	**< 0.001**
Infarct volume, ml	7 (2–18)	10 (4–23)	0.146
MVO, n (%)	16 (15)	81 (33)	**< 0.001**

### Binary logistic regression

In univariable analyses, BMI (metric and dichotomized) was a significant predictor of MVO (metric: odds ratio (OR): 1.3, 95% confidence interval (CI): 1.0-1.5, *p* = 0.031; dichotomized: OR: 2.8, 95%CI: 1.6–5.1, *p* < 0.001). In several multivariable models containing clinical parameters only (model I), CMR parameters (model II) or a mix of them including peak troponin T levels (model III), a BMI > 25.6 kg/m² remained a significant predictor of MVO (OR: ≥2.5, *p* < 0.020). When setting the threshold for dichotomization at 25 kg/m² (i.e. non-overweight vs. overweight), an increased BMI was also significant in all three models (OR: >2.4, *p* < 0.040). When replacing dichotomized BMI with BMI as a continuous variable, it remained significant in model I (OR: 1.3, 95%CI: 1.0-1.6, *p* = 0.018), but not in models II and III (*p* > 0.100) A detailed view of logistic regression results is shown in Table 3.

**Table 3:** Binary logistic regression analysis for the dependent variable microvascular obstruction. Metric variables are stratified into quartiles. *BMI: body mass index; CMR: cardiac magnetic resonance imaging; EDV: end-diastolic-volume*,* EF: ejection fraction; ESV: end-systolic volume; hs: high-sensitive; LV: left ventricular*

Parameter	Odds Ratio	95% Confidence Interval	*p*-value
¬ Univariable			
BMI, kg/m²	1.3	1.0-1.5	**0.031**
BMI > 25.6 kg/m²	2.8	1.6–5.1	**< 0.001**
Age, years	0.7	0.5-0.8	**< 0.001**
Male sex	2.0	1.1–3.6	**0.027**
Diabetes	0.5	0.3-0.9	**0.023**
TIMI Flow 0 (no-reflow)	4.3	2.6-7.0	**< 0.001**
Peak hs-Troponin T, ng/ml	2.5	1.9-3-3	**< 0.001**
LV-EF, %	0.6	0.5-0.8	**< 0.001**
LV-EDV, ml	1.5	1.2–1.9	**< 0.001**
LV-ESV, ml	1.7	1.4–2.1	**< 0.001**
LV myocardial mass, g	1.7	1.4–2.2	**< 0.001**
Infarct Volume, ml	3.3	2.4 (4.3)	**< 0.001**
¬ **Multivariable**			
***- Model I (Clinical)***			
BMI > 25.6 kg/m²	2.7	1.5–4.9	**0.002**
Age, years	0.7	0.6-0.9	**0.005**
Male sex	1.6	0.8-2.9	0.165
Diabetes	0.6	0.3-1.1	0.072
***- Model II (CMR)***			
BMI > 25.6 kg/m²	3.0	1.5–5.8	**0.002**
LV-EF, %	0.8	0.6 − 1.0	**0.036**
Infarct volume, ml	3.0	2.2–4.1	**< 0.001**
***- Model III (Mixed)***			
BMI > 25.6 kg/m²	2.5	1.2–5.3	**0.019**
Age, years	0.7	0.5–0.9	**0.013**
TIMI Flow 0 (no-reflow)	2.9	1.5–5.5	**0.002**
Peak hs-Troponin T, ng/ml	1.5	1.1–2.1	**0.013**
LV-EF, %	0.7	0.6-1.0	0.052
Infarct volume, ml	2.3	1.6–3.3	**< 0.001**

## Discussion

To the best of our knowledge, this study is the first to show the influence of being overweight as defined by an increased BMI on the development of MVO in NSTEMI patients. In summary, our results were as follows: (a) patients with MVO had a significantly higher BMI; (b) dichotomized at a threshold of 25.6 kg/m², patients above that value were significantly more often affected by MVO; (c) a BMI above that value was significantly associated with the development of MVO after NSTEMI, independent of traditional cardiovascular risk factors.

In general, overweight has been shown to dramatically increase the risk of cardiovascular disease, particularly of myocardial infarction [9, 17]. While the exact pathophysiology of this connection is not fully understood yet, local and systemic inflammatory processes seem to be key factors here [18]. Furthermore, inflammatory processes have also been linked to the occurrence of MVO in STEMI [19, 20], and even to a potential persistence of MVO [21]. As such, it could be assumed that overweight also triggers MVO development. However, several studies have suggested that the opposite is the case: in 2022, a study on 225 STEMI patients has shown a lower risk for MVO in those with a BMI > 28 kg/m² [7]. Another study on a similarly sized patient group also described a risk reduction for MVO with growing BMI in STEMI [22]. Conversely, a large study including more than 2,200 STEMI patients undergoing PCI did not find any association between BMI and the development of MVO [23], thus challenging the so-called “obesity paradox” in myocardial infarction [24]. Nevertheless, there are limited data available investigating the impact of BMI on MVO in NSTEMI patients. Still, the “obesity paradox” has also been proposed in NSTEMI, suggesting that those patients could benefit from overweight in terms of mortality [10]. This possible beneficial effect is in contrast to our data, showing that NSTEMI patients with overweight, especially those with a BMI above 25.6 kg/m² (i.e., at least mildly increased) were more than twice as often affected by MVO than those with a lower BMI and, by tendency, also had larger infarcts. While in this study, an ideal cutoff of 25.6 kg/m² (Youden-index) was used, probably a more intuitive threshold would be 25 kg/m², representing the cutoff between a normal BMI and overweight. As depicted in Fig. 2, after stratifying the patient cohort into BMI classes, the highest MVO prevalence was in the group with mildly increased BMI, while gradually declining with growing BMI, possibly hinting that a very high BMI could again be less prone to MVO development. However, this subgroup included only 28 patients, and the apparent decrease did not reach statistical significance. Therefore, the observed non-linear pattern should be interpreted with caution. While there is a plethora contributing to microvascular injury in acute myocardial infarction, our study was well in line with literature concerning other reported factors frequently concomitant to MVO, such as infarct size, TIMI flow, increased troponin levels or impaired functional parameters [4, 21, 25–27]. Diabetes has been associated with MVO in several previous studies [28, 29]. Moreover, according to a study by Giusca et al., myocardial scar derived from CMR is a stronger predictor of adverse outcomes in patients with diabetes than in those without [30]. Interestingly, in the present study diabetes showed an inverse association with MVO and was also a “protective” factor in univariable analysis; however, it did not remain significant in multivariable analysis. Besides, patients with MVO in our study were significantly less often female. Although available data are scarce, prior studies did not show an association between sex and MVO [31], nor divergent outcomes of microvascular injury regarding sex [32]. Patients with MVO in our study were significantly younger, which is contrary to current literature [33]. This, however, can probably be attributed to younger patients also showing larger infarcts in the current study, which in return predisposes for MVO [21, 34]. The latter is also in line with this current study, where infarct size remained a significant marker of MVO in multivariable models. Lastly, despite several factors contributing to the development of MVO have been well described and reported in the past, overweight might still play an additional role in the development of microvascular injury, among other factors probably due to its link to generalized inflammation [18]. In this regard, even mildly increased BMI might contribute to elevated systemic inflammatory markers [35]. Another decisive factor associated with MVO is a low angiographic TIMI flow pre- and post-PCI [36]; as such, patients with an increased BMI showed less often a TIMI flow 3 after intervention, indicating less effective revascularization. This, again, is in line with recent studies on STEMI and acute coronary syndrome [37, 38]. Further, while the presence of MVO is primarily associated with STEMI rather than NSTEMI, this finding might be related to a high proportion of culprit lesions located in the left circumflex artery (35%). Complete occlusions might have been more frequent in the left circumflex than in the left anterior descending coronary artery [39]. Then, where in STEMI at least mild overweight seemed to have a protective effect on MVO development, our data suggest that in NSTEMI the opposite phenomenon could be present. While a final explanation on this association cannot be given with certainty, a possible explanation could lie in a more nuanced pathophysiology of NSTEMI as opposed to an often rather paroxysmal nature of STEMI: in the prior group, there might be a more modulated systemic immune answer that could possibly lead to an exacerbation of long-term microvascular and inflammatory disease and, thus, may lead to a higher susceptibility to microvascular damage.

### Limitations

This study bears several limitations. First, comprehensive lab analyses were not fully available in all patients; however, including other parameters such as C-reactive protein or natriuretic peptides into analyses could have corroborated this study’s conclusions; especially, a broader availability of the prior could have potentially highlighted an association of BMI and inflammation. Second, CMR protocols were focused on late enhancement and functional analysis; yet wider protocols including mapping sequences would have added additional value to this current investigation. Third, the characterization of intramyocardial iron (e.g., by using iron-sensitive T2* mapping sequences) would have provided a more multidimensional view on microvascular damage [32]. Fourth, BMI was used as a surrogate for overweight; it does not differentiate between fat distribution or muscle mass, which may have distinct pathophysiological implications. Lastly, the small number of included patients might also play a role in the validity of this current analysis; larger studies to assess this association are desirable.

## Conclusion

This study showed that overweight increases the risk of MVO occurrence in NSTEMI patients. Thereby, overweight and especially a BMI above a threshold of 25.6 kg/m² could have been shown to be significant predictors for the development of MVO, independent of traditional cardiovascular risk markers. These findings challenge the so-called “obesity paradox”, at least in NSTEMI patients. However, further studies are needed to corroborate this association, and outcome studies investigating the clinical impact are needed.

## Data Availability

All data supporting the findings of this study are available within the paper or on reasonable request from the corresponding authors.

## Abbreviations

BMI: Body Mass Index
CI: Confidence Interval
CMR: Cardiovascular Magnetic Resonance Imaging
EF: Ejection Fraction
IQR: Interquartile Range
LV: Left Ventricle/Ventricular
MVO: Microvascular Obstruction
NSTEMI: Non-ST-Elevation Myocardial Infarction
PCI: Percutaneous Coronary Intervention
ROC: Receiver Operating Characteristic
STEMI: ST-Elevation Myocardial Infarction
TIMI: Thrombolysis in Myocardial Infarction

## References

[1] Konijnenberg LSF, Damman P, Duncker DJ et al (2020) Pathophysiology and diagnosis of coronary microvascular dysfunction in ST-elevation myocardial infarction. Cardiovasc Res 116:787–805 31710673 10.1093/cvr/cvz301 PMC7061278

[2] Carrick D, Haig C, Ahmed N et al (2016) Myocardial hemorrhage after acute reperfused ST-Segment-Elevation myocardial infarction: relation to microvascular obstruction and prognostic significance. Circ Cardiovasc Imaging 9:e004148 26763281 10.1161/CIRCIMAGING.115.004148 PMC4718183

[3] Brado J, Schmitt R, Hein M et al (2025) Predicting MRI-diagnosed microvascular obstruction and its long-term impact after acute myocardial infarction. Clin Res Cardiol. Epub ahead of print. 10.1007/s00392-025-02709-1 40622620

[4] Reinstadler SJ, Stiermaier T, Fuernau G et al (2016) The challenges and impact of microvascular injury in ST-elevation myocardial infarction. Expert Rev Cardiovasc Ther 14:431–443 26794717 10.1586/14779072.2016.1135055

[5] Ndrepepa G, Tiroch K, Fusaro M et al (2010) 5-year prognostic value of no-reflow phenomenon after percutaneous coronary intervention in patients with acute myocardial infarction. J Am Coll Cardiol 55:2383–2389 20488311 10.1016/j.jacc.2009.12.054

[6] Byrne RA, Rossello X, Coughlan JJ et al (2023) 2023 ESC guidelines for the management of acute coronary syndromes. Eur Heart J 44:3720–3826 37622654 10.1093/eurheartj/ehad191

[7] Lan DH, Zhang Y, Hua B et al (2022) Impact of obesity on microvascular obstruction and area at risk in patients after ST-Segment-Elevation myocardial infarction: A magnetic resonance imaging study. Diabetes Metab Syndr Obes 15:2207–2216 35923250 10.2147/DMSO.S369222 PMC9342698

[8] Jiang L, Shi K, Guo YK et al (2020) The additive effects of obesity on myocardial microcirculation in diabetic individuals: a cardiac magnetic resonance first-pass perfusion study. Cardiovasc Diabetol 19:52 32375795 10.1186/s12933-020-01028-1 PMC7201945

[9] Madala MC, Franklin BA, Chen AY et al (2008) Obesity and age of first non-ST-segment elevation myocardial infarction. J Am Coll Cardiol 52:979–985 18786477 10.1016/j.jacc.2008.04.067

[10] Buettner HJ, Mueller C, Gick M et al (2007) The impact of obesity on mortality in UA/non-ST-segment elevation myocardial infarction. Eur Heart J 28:1694–1701 17576661 10.1093/eurheartj/ehm220

[11] de Waha S, Eitel I, Desch S et al (2013) Thrombus aspiration in thrombus containing culprit lesions in Non-ST-Elevation myocardial infarction (TATORT-NSTEMI): study protocol for a randomized controlled trial. Trials 14:110 23782681 10.1186/1745-6215-14-110 PMC3748830

[12] Thiele H, de Waha S, Zeymer U et al (2014) Effect of aspiration thrombectomy on microvascular obstruction in NSTEMI patients: the TATORT-NSTEMI trial. J Am Coll Cardiol 64:1117–1124 25212646 10.1016/j.jacc.2014.05.064

[13] McEntegart MB, Kirtane AJ, Cristea E et al (2012) Intraprocedural thrombotic events during percutaneous coronary intervention in patients with non-ST-segment elevation acute coronary syndromes are associated with adverse outcomes: analysis from the ACUITY (Acute catheterization and urgent intervention triage Strategy) trial. J Am Coll Cardiol 59:1745–1751 22575311 10.1016/j.jacc.2012.02.019

[14] Hong YJ, Jeong MH, Choi YH et al (2011) Impact of plaque components on no-reflow phenomenon after stent deployment in patients with acute coronary syndrome: a virtual histology-intravascular ultrasound analysis. Eur Heart J 32:2059–2066 19228713 10.1093/eurheartj/ehp034 PMC3155758

[15] Wang TY, Zhang M, Fu Y et al (2009) Incidence, distribution, and prognostic impact of occluded culprit arteries among patients with non-ST-elevation acute coronary syndromes undergoing diagnostic angiography. Am Heart J 157:716–723 19332201 10.1016/j.ahj.2009.01.004

[16] Clinical Guidelines on the (1998) Identification, Evaluation, and treatment of overweight and obesity in Adults–The evidence Report. National institutes of health. Obes Res 6(Suppl 2):51S–209S 9813653

[17] Powell-Wiley TM, Poirier P, Burke LE et al (2021) Obesity and cardiovascular disease: A scientific statement from the American heart association. Circulation 143:e984–e1010 33882682 10.1161/CIR.0000000000000973 PMC8493650

[18] Wannamethee SG, Whincup PH, Rumley A, Lowe GD (2007) Inter-relationships of interleukin-6, cardiovascular risk factors and the metabolic syndrome among older men. J Thromb Haemost 5:1637–1643 17596140 10.1111/j.1538-7836.2007.02643.x

[19] Montone RA, La Vecchia G (2021) Interplay between inflammation and microvascular obstruction in ST-segment elevation myocardial infarction: the importance of velocity. Int J Cardiol 339:7–9 34311010 10.1016/j.ijcard.2021.07.041

[20] Holzknecht M, Tiller C, Reindl M et al (2021) C-reactive protein velocity predicts microvascular pathology after acute ST-elevation myocardial infarction. Int J Cardiol 338:30–36 34147553 10.1016/j.ijcard.2021.06.023

[21] Troger F, Pamminger M, Poskaite P et al (2025) Clinical impact of persistent microvascular obstruction in CMR after reperfused STEMI. Circ Cardiovasc Imaging 18(6) :e017645 10.1161/CIRCIMAGING.124.017645 40357554

[22] He Y, Li X, Gao J, Li K (2024) Influence of obesity on microvascular obstruction and the myocardial area at risk in patients with ST-segment elevation myocardial infarction. Am J Transl Res 16:6736–6744 39678541 10.62347/PYEF1488 PMC11645562

[23] Shahim B, Redfors B, Chen S et al (2020) BMI, infarct Size, and clinical outcomes following primary PCI: Patient-Level analysis from 6 randomized trials. JACC Cardiovasc Interv 13:965–972 32327093 10.1016/j.jcin.2020.02.004

[24] Bucholz EM, Beckman AL, Krumholz HA, Krumholz HM, conducted, DBwawtYSoMaYSoPHdttttww (2016) Excess weight and life expectancy after acute myocardial infarction: The obesity paradox reexamined. Am Heart J. ;172:173 – 81 10.1016/j.ahj.2015.10.024 PMC5097250 26856230

[25] Bodi V, Gavara J, Lopez-Lereu MP et al (2023) Impact of persistent microvascular obstruction late after STEMI on adverse LV remodeling: A CMR study. JACC Cardiovasc Imaging 16:919–930 37052556 10.1016/j.jcmg.2023.01.021

[26] Mayr A, Klug G, Reindl M et al (2022) Evolution of myocardial tissue injury: A CMR study over a decade after STEMI. JACC Cardiovasc Imaging 15:1030–1042 35680211 10.1016/j.jcmg.2022.02.010

[27] van Kranenburg M, Magro M, Thiele H et al (2014) Prognostic value of microvascular obstruction and infarct size, as measured by CMR in STEMI patients. JACC Cardiovasc Imaging 7:930–939 25212799 10.1016/j.jcmg.2014.05.010

[28] Husser O, Bodi V, Sanchis J et al (2013) Predictors of cardiovascular magnetic resonance-derived microvascular obstruction on patient admission in STEMI. Int J Cardiol 166:77–84 22018514 10.1016/j.ijcard.2011.09.083

[29] Jaffe R, Charron T, Puley G, Dick A, Strauss BH (2008) Microvascular obstruction and the no-reflow phenomenon after percutaneous coronary intervention. Circulation 117:3152–3156 18559715 10.1161/CIRCULATIONAHA.107.742312

[30] Giusca S, Kelle S, Nagel E et al (2016) Differences in the prognostic relevance of myocardial ischaemia and Scar by cardiac magnetic resonance in patients with and without diabetes mellitus. Eur Heart J Cardiovasc Imaging 17:812–820 26358695 10.1093/ehjci/jev220

[31] Langhans B, Ibrahim T, Hausleiter J et al (2013) Gender differences in contrast-enhanced magnetic resonance imaging after acute myocardial infarction. Int J Cardiovasc Imaging 29:643–650 23053858 10.1007/s10554-012-0132-3

[32] Lechner I, Reindl M, Stiermaier T et al (2024) Clinical outcomes associated with various microvascular injury patterns identified by CMR after STEMI. J Am Coll Cardiol 83:2052–2062 38777509 10.1016/j.jacc.2024.03.408

[33] Park IH, Cho HK, Oh JH et al (2021) Old age and myocardial injury in ST-Segment elevation myocardial infarction. Am J Med Sci 362:592–600 34563496 10.1016/j.amjms.2021.06.023

[34] Doherty DJ, Sykes R, Mangion K, Berry C (2021) Predictors of microvascular reperfusion after myocardial infarction. Curr Cardiol Rep 23:21 33624185 10.1007/s11886-021-01442-1 PMC7902326

[35] Cohen E, Margalit I, Shochat T, Goldberg E, Krause I (2021) Markers of chronic inflammation in overweight and obese individuals and the role of gender: A Cross-Sectional study of a large cohort. J Inflamm Res 14:567–573 33658829 10.2147/JIR.S294368 PMC7920597

[36] Schaaf MJ, Mewton N, Rioufol G et al (2016) Pre-PCI angiographic TIMI flow in the culprit coronary artery influences infarct size and microvascular obstruction in STEMI patients. J Cardiol 67:248–253 26116981 10.1016/j.jjcc.2015.05.008

[37] Niedziela J, Hudzik B, Niedziela N et al (2014) The obesity paradox in acute coronary syndrome: a meta-analysis. Eur J Epidemiol 29:801–812 25354991 10.1007/s10654-014-9961-9 PMC4220102

[38] Lee SH, Jeong MH, Kim JH et al (2018) Influence of obesity and metabolic syndrome on clinical outcomes of ST-segment elevation myocardial infarction in men undergoing primary percutaneous coronary intervention. J Cardiol 72:328–334 29709405 10.1016/j.jjcc.2018.03.010

[39] Feistritzer HJ, Nanos M, Eitel I et al (2020) Determinants and prognostic value of cardiac magnetic resonance imaging-derived infarct characteristics in non-ST-elevation myocardial infarction. Eur Heart J Cardiovasc Imaging 21:67–76 31518417 10.1093/ehjci/jez165

